# Evaluating the products of low power, sealed vessel microwave pyrolysis of microalgae

**DOI:** 10.3389/fchem.2026.1735803

**Published:** 2026-03-12

**Authors:** Jolyon J. Glynn, Ewan. D. Ward, Donal McGee, Avtar S. Matharu

**Affiliations:** 1 Department of Chemistry, Green Chemistry Centre of Excellence, University of York, York, United Kingdom; 2 AlgaeCytes Limited UK, Sandwich, United Kingdom

**Keywords:** biochar, bio-oil, microalgae, microwave pyrolysis, nannochloropsis

## Abstract

The products of low power, sealed vessel microwave pyrolysis of a proprietary microalga, ALG01, similar to *Eustigmatophyceae*, were evaluated at fixed power (50 W, 100 W and 150 W). The afforded biochars were characterised by CHNS, ATR-IR, SS 13C NMR, TGA and SEM, whilst the bio-oils were characterised by CHNS, 1H and 13C NMR, GC-MS/FID and ATR-IR. The bio-oils were all rich in fatty acids, although with increased microwave power, these long chain fatty acids experienced cracking. The higher heating values (HHVs) of the bio-oils was calculated, with the 50 W bio-oil attaining the highest HHV (35.04 ± 2.40 MJ/kg). Preliminary copper(II) adsorption studies were performed on the biochars, with activated carbon (AC) used as a control. The biochar produced at 150 W adsorbed the highest amount of copper(II) when dosed at both 1 mg/mL and 10 mg/mL of biochar (110.00 ± 4.83 and 31.26 ± 2.63 mg/g, respectively). Thus, the use of low power as opposed to high power and/or high temperature conventional pyrolysis yields interesting products that can be used as potential platform chemicals, adsorbents for critical element recovery and as a potential solid biofuel. However, the latter application is limited because the HHV is similar to that of low-grade coal and has a relatively high ash content.

## 1 Introduction

Microalgae are an excellent third-generation biomass feedstock. They thrive in a variety of environments, from fresh water closed-loop bioreactors, salt water open lakes or even wastewater mediums; allowing prime arable land to remain unaffected (Chua et al., 2017; Yang et al., 2019). Moreover, some microalgae may grow up to 50 times faster than the fastest growing terrestrial plant (switch grass) (Li and Et, 2008). Therefore, microalgae can assimilate up to twice their mass in CO_2_ daily, implying that 1 ha of cultivated algae could sequester approximately 365 tonnes of CO_2_ annually (Sukarni, 2014). Furthermore, microalgae are a source of high-value compounds, such as lipids, pigments, carbohydrates, and proteins, which have potential applications in nutraceuticals, pharmaceuticals, and cosmetics (Chua et al., 2017; Li and Et, 2008).

ALG01 is a proprietary strain of microalgae, which is related to *Nannochloropsis. Nannochloropsis* is a spherical (or ovoid) shaped unicellular marine algae (2–5 µm in diameter) and part of the Eustigmatophycae family (Chua et al., 2017). Eustigmatophytes contain exclusively chlorophyll a, and in *Nannochloropsis*, this is enclosed within a single chloroplast alongside violaxanthin, the predominant carotenoid (Dolganyuk et al., 2020). The cell wall of *Nannochloropsis* is predominantly cellulose (75%), with an external Algaenan layer (Ribeiro et al., 2022). Algaenan comprises long chain aliphatic carbons with ether cross links, resulting in a highly resistant material to both chemical (acid/alkali) or physical (organic/aqueous dissolution) processing (Chua et al., 2017; Surkani et al., 2014). *Nannochloropsis* has been of interest as a feedstock for biorefineries due to its high lipid content (∼30%). Microalgal lipids are typically applied to biofuel industries (Sivaramakrishnan et al., 2022), however, they are high in polyunsaturated fatty acids (PUFA) content, such as eicosapentaenoic acid (EPA, 2%–6%) and, thus, *Nannochloropsis* oils would be vulnerable to oxidation without fractionation (Helwani et al., 2023). Fortunately, EPA has been shown to have many human health benefits, such as cardiovascular health (Mozaffarian et al., 2012). In fact, there is an increasing demand for EPA and docosahexaenoic acid (DHA) omega-3 fatty acids: estimated at approximately 390-780 kilotonnes annually by 2050 (USA Institute of Medicine RDI: 0.1–0.2 g) (United Nations, 2024). Microalgae is a key source of vegan EPA that is eco-friendly and avoids overfishing, however, following EPA extraction there is still a significant quantity of biomass residues. Further processing is often required to valorise these residues, but unfortunately extracting the high-value proteins, pigments, or carbohydrates can be economically unfeasible due to the challenge of overcoming the rigid cell walls (Lee et al., 2017).

Pyrolysis is a valorisation process in which biomass is heated under an inert atmosphere to produce high-value biochar, bio-oil and biogas. The products of pyrolysis via conventional heating can be manipulated using the heating rate. The 3 major forms of traditional pyrolysis include: slow pyrolysis, fast pyrolysis and flash pyrolysis. Slow pyrolysis, as implied, uses a gradual heating rate followed by a prolonged isothermal hold and favours biochar production. Fast pyrolysis heats more rapidly, enabling increased yields of bio-oil due to the release of pyrolytic vapours. Flash pyrolysis employs even higher heating rates (up to 10,000 °C/s) and yields primarily bio-oil. There are a few downsides to conventional pyrolysis of solids, in particular high energy requirements, often due to heat loss, and conduction-limited heating often resulting in uneven thermal gradients (Lin et al., 2023). Microwave pyrolysis offers a greener alternative to conventional pyrolysis as, due to dielectric heating, the sample is heated throughout, decreasing the required time to reach temperature and reducing the required temperature for reactions to occur (Lin et al., 2023).

Recent research on microwave pyrolysis of microalgae has focused on using high power microwaves; 300–900 W (Mohit et al., 2023), 250–950 W (Beneroso et al., 2013), 500–1250 W (Du et al., 2011), 750–2250 W (Hu et al., 2012), 600 W (Qadariyah et al., 2021), 700 W (Gümüş et al., 2022), 1200 W (Nawaz et al., 2024). Du et al. (2011) demonstrated that increasing microwave power alters the three-phase product distribution, with biogas yields rising by 10% between 500 W and 1250 W. Furthermore, Mohit et al. (2023) reported that lower power (300–400 W) and shorter reaction times significantly increased biochar yields (from 10% to 20% at 800–900 W to 55%–70%). These variations in power, temperature, and reaction time also dictate composition of the pyrolysis products. High power conditions generally enhance biochar carbon content and increase H_2_ and CH_4_ biogas concentrations while reducing CO_2_ levels (Du et al., 2011; Mohit et al., 2023). To optimise energy efficiency and product selectivity, catalysts and heat adsorbers such as calcium oxide (Qadariyah et al., 2021), silicon carbide (Nawaz et al., 2024), activated carbon (Hu et al., 2012) and biochar (Du et al., 2011) are commonly employed. These materials facilitate rapid heating, shorter reaction times, and provide active surfaces for reactions such as cracking.

While current research establishes the role of high-power or catalyst-assisted processes, little attention has been given to low-power microwave pyrolysis. In this work, we investigate microwave-assisted pyrolysis of microalgae at 50–150 W in a closed system. Unlike conventional open-vessel microwave reactors that rely on a continuous gas purge, the closed configuration allows for pressure build-up, a variable whose influence has not been previously reported in this area, to the authors’ knowledge. A proprietary strain of industrially defatted microalgae: ALG01, related to *Nannochloropsis*, was subject to sealed vessel microwave pyrolysis at set powers of 50 W, 100 W and 150 W, without the presence of a catalyst. The resultant mass was washed with ethyl acetate to separate biochar from bio-oil. Biochars were characterised by ATR-IR, TGA, CHNS, SEM and SS 13C NMR, and a preliminary copper(II) adsorption study was conducted as a potential application. Bio-oil were characterised by ATR-IR, CHNS, GC-MS, H NMR and 13C NMR.

## 2 Materials and methods

### 2.1 Materials, ALG01

Defatted algal biomass was provided by AlgaeCytes Ltd UK which had undergone their lipid transesterification extraction process (Table 1). Samples were left to dry in a 105 °C oven overnight to ensure dryness.

**TABLE 1:** Characteristics of defatted ALG01 biomass.

Proximate analysis^a^ (wt.%)		Elemental analysis^b^ (wt.%)		Composition analysis^a^ (wt.%)	
Moisture	4.1 ± 0.1	C	42.14 ± 0.12	Lipid^d^	3.3 ± 0.1
Volatile	73.3 ± 0.2	H	5.88 ± 0.07	Protein^e^	46.5 ± 0.6
Fixed carbon	22.3 ± 0.6	N	7.93 ± 0.24	Carbohydrate^f^	26.8 ± 4.7
Ash	16.46 ± 1.57	O^c^	27.2	Acid-insoluble^f^	13.2 ± 1.5

^a^Wet basis.

^b^Dry basis.

^c^By difference O(%) = 100-C-H-N-Ash.

^d^Heptane extraction.

^e^6.25x N content.

^f^Acid-digestion sugar analysis.

### 2.2 Microwave pyrolysis

Microwave pyrolysis was carried out using a CEM Discover microwave. Dried ALG01(0.5 g) was weighed into a glass microwave tube (10 cm length x 1 cm internal diameter) and capped (PTFE). Pyrolysis was carried out at a fixed power (50 W, 100 W or 150 W), with a safe operating temperature and pressure set at 280 °C and 300 psi, respectively. The total run time was 20 min. Each run was performed in triplicate. Upon completion, ethyl acetate (10 mL) was added to the microwave tube to extract any bio-oil. The mixture was filtered to separate the organic extract from the char. The organic extract was dried (MgSO_4_), filtered and evaporated *in vacuo* to afford the resultant bio-oil, whereas, the biochar was allowed to air dry overnight to constant weight. The bio-oil, biochar and bio-gas yields were calculated using the following equations:

$${Yield}_{biochar}\;\left(\%\right)=\left(\frac{{Mass}_{biochar}}{{Mass}_{ALGO1}}\right)\times 100\%$$

$${Yield}_{bio\;oil}\;\left(\%\right)=\left(\frac{{Mass}_{bio\;oil}}{{Mass}_{ALGO1}}\right)\times 100\%$$

$${Yield}_{biogas}\;\left(\%\right)={Mass}_{ALGO1}-{Mass}_{bio\;oil}-{Mass}_{biochar}$$

### 2.3 Higher heating value (HHV)

The HHV was estimated according to Channiwala and Parikh (2002) shown below.

$$HHV\left(MJ/kg\right)=0.3491\cdot C\;\left(wt.\%\right)+1.1783\cdot H\;\left(wt.\%\right)+0.1005\cdot S\;\left(wt.\%\right)-0.1034\cdot O\left(wt.\%\right)-0.0151\cdot N\left(wt.\%\right)-0.0211\cdot A\left(wt.\%\right)$$

Where the values of **C**, **H**, **N** and **S** were obtained directly from CHNS analysis, the value for **A** (Ash content) was determined by TGA and the value of **O** was determined by the following equation:

$$O\left(wt.\%\right)=100\%-C\left(wt.\%\right)-H\left(wt.\%\right)-S\left(wt.\%\right)-N\left(wt.\%\right)-A\left(wt.\%\right)$$

### 2.4 Thermogravimetric analysis (TGA)

The biochar (∼30 mg) samples were run on a Netzsch STA 409 under nitrogen (100 mL/min), with a ramp of 10 °C/min from 25 °C to 650 °C. The ash content was determined by an isothermal hold at 650 °C for 30 min whilst oxygen was introduced. The data was processed using Origin 2018™ software.

### 2.5 Elemental analysis (CHNS)

Samples for CHNS were submitted as an internal service for analysis on a Thermo Scientific™ Flash*Smart*™ Elemental Analyser.

### 2.6 Scanning electron microscope (SEM)

Imaging was carried out on a JEOL JSM7800F SEM at the University of York Jeol Nanocentre. Samples were placed onto aluminium stubs with carbon tape before being sputtered with copper (∼10 nm).

### 2.7 Solid state 13 carbon cross polarisation magic spinning angel nclear magnetic resonance (SS 13C CPMAS NMR)

SS 13C CPMAS NMR was carried out on a Brucker 400 MHz Avance III HD spectrometer using a 2.5 mm rotor spun at 20 kHz. A total number of 16,384 scans were used with a D1 of 4 s. The data was then processed in MestReNova software.

### 2.8 1H/13C NMR

1H (400 MHz) and 13C NMR (101 MHz) was run on a Jeol ECS 400 MHz NMR spectrometer with 8 and 2048 scans used, respectively. ∼15 mg of oil was dissolved in 0.8 mL CDCl_3_.

### 2.9 GC-FID

The Agilent Technologies HP 6890 gas chromatograph was used, with a flame ionisation detector, fitted with a Rxi-5HT capillary column (30 m, 250 mm × 0.25 mm nominal, max temperature 400 °C). Hydrogen was used as the carrier gas, at a flow rate of 10 mL/min, a split rate of 5:1 and a 1 µL injection. The oven’s starting temperature was 50 °C, which was increased to 300 °C at a rate of 30 °C/min, where it was held for 5 min. The total run time was 13.3 min. Injection temperature and the detector temperature were both 250 °C.

### 2.10 GC-MS

The Agilent Technologies 7890B gas chromatograph was used, joined with a Clarus 560 MS. The GC was fitted with a Rxi-5HT capillary column (30 m, 250 mm × 0.25 mm nominal, max temperature 400 °C). Helium was used as the carrier gas, at a flow rate of 10 mL/min, a split rate of 5:1 and a 1 µL injection. The oven’s starting temperature was 50 °C, which was increased to 300 °C at a rate of 30 °C/min, where it was held for 5min. The total run time was 13.3min. Injection temperature and the detector temperature were both 250 °C. Mass spec scans were run over 40 m/z to 500m/z with a solvent delay of 3 min. The total run time was 13.33 min. NIST library version 2.2 was used to analyse the MS data.

### 2.11 ATR-IR

ATR-IR was conducted using a Perkin-Elmer FT-IR Spectrometer. Before placing the sample on the crystal, it was cleaned with isopropyl alcohol and tissues. The spectra were recorded between 450 and 4,000 cm^-1^ (16 scans). The data was then processed using Origin 2018™ software.

### 2.12 Copper(II) adsorption studies

Standard solutions of copper nitrate trihydrate (3,500–100 mg/L) were prepared from a stock solution of copper nitrate trihydrate (5,000 mg/1 L). The UV-vis spectrum (Shimadzu UV-vis 1800, 900–400 nm) of these solutions was recorded to create a calibration curve (R^2^ = 0.9993, see ESI).

Prior to copper adsorption studies, the biochars were washed in hot distilled water, filtered and rewashed with hot water, acetone and then dried (105 °C) overnight. The dried biochar (1 or 10 mg/mL) was added to a solution of copper nitrate trihydrate (2,500 mg/L, 10 mL), in a 25 mL conical flask, stoppered and shook (200 rpm) in a thermostatically heated waterbath at 30 °C. Thereafter, the mixture was syringe filtered through a regenerated cellulose filter (13 mm diameter x 0.22 μm pore width) into a UV cuvette and its UV-vis absorbance spectrum recorded. The copper(II) concentration was determined at 810 nm using the calibration curve. This procedure was repeated in triplicate.

## 3 Results and discussion

### 3.1 Microwave pyrolysis of ALG01

**FIGURE 1:**
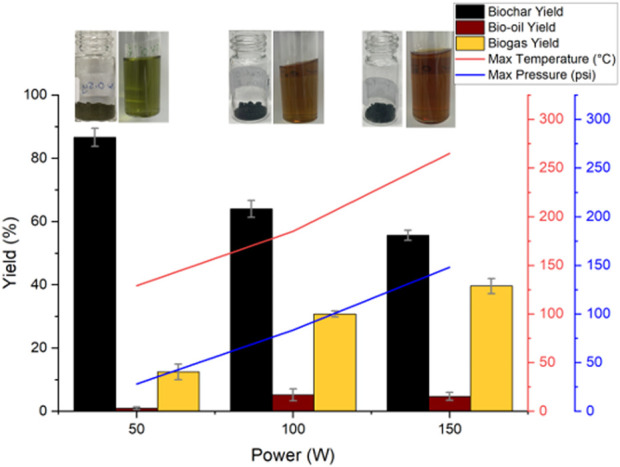
ALG01 Biochar, bio-oil and biogas yields following fixed power microwave pyrolysis (50–150 W) and the resulting maximum temperature and pressure measured. Inset are images of the biochar and bio-oil samples (bio-oils diluted in ethyl acetate to accentuate their colour).

The biochar, bio-oil and biogas yields are depicted in Figure 1, alongside the maximum temperature and pressure achieved within the reaction vessel. Increasing microwave power led to an increase in the maximum temperature and pressure, with the 150 W run achieving 265 °C ± 10 °C and 148 ± 34 psi, whereas the 50 W run only reached 129 °C ± 10 °C and 28 ± 4 psi. Tukey’s HSD post hoc test showed there was a significant difference in maximum achieved temperature between all runs whilst maximum achieved pressure was only significantly different between the 50 W and 150 W runs (Supplementary Table S1). The reaction temperature for this system at 150 W is only ∼40 °C cooler than values reported in the literature under comparable conditions but using graphite as an adsorbent (Cho et al., 2009). However, it is likely that the recorded temperature is lower than the actual internal temperature. Since microalga is a poor microwave absorber, carbonaceous hot spots form, leading to uneven microwave heating. Moreover, the CEM Discover microwave is equipped with a bottom mounted IR temperature sensor, which predominantly measures the vessel wall temperature. Therefore, potential hot spots within the bulk or towards the top of the sample may not be accurately reflected in the recorded temperature. Furthermore, the temperature of the vapour in the top of the microwave tube is also not accounted for. Ideally, in combination with the external IR temperature sensor, an internal thermocouple would be used, although this is difficult to implement in a closed vessel reactor (Bartoli et al., 2019).

As microwave power increased, the biochar yield decreased from 86.57% ± 3.25% (50 W) to 55.67% ± 1.57% (150 W), whilst the biogas yield increased from 12.46% ± 2.82% (50 W) to 39.61% ± 2.38% (150 W), in alignment with literature (Du et al., 2011; Mohit et al., 2023). Interestingly, the bio-oil yield increased from 0.98% ± 0.50% to 4.70% ± 1.29% between 50 W and 100 W, however, then plateaued between 100 W and 150 W. Tukey’s HSD post hoc test showed that the 50 W run exhibited a significant difference in both biochar and bio-oil yield whilst no significant difference was observed between the 100 W and 150 W biochar and bio-oil yields. The bio-gas yield was significantly different between all 3 runs (Supplementary Table S2). Upon visual inspection, the biochar from the 50 W run was similar in appearance to the starting material, which likely implies thermally-induced degradation was minimal. The 100 W and 150 W runs both appeared to be increasingly black and brittle suggesting the materials had begun to pyrolyse. The biomass’ chlorophyll content is not yet degraded in the 50 W run as the bio-oil produced was still bright green. However, the bio-oils from the 100 W and 150 W runs were both dark brown indicating the formation of Maillard products above 160 °C (Quintain et al., 2013). All of the pyrolyses produced a very strong and sticky odour, with the higher power runs achieving a stronger and more pungent smell.

### 3.2 Biochar analysis

#### 3.2.1 Elemental analysis (CHNS), ash content, and higher heating value (HHV)

**FIGURE 2:**
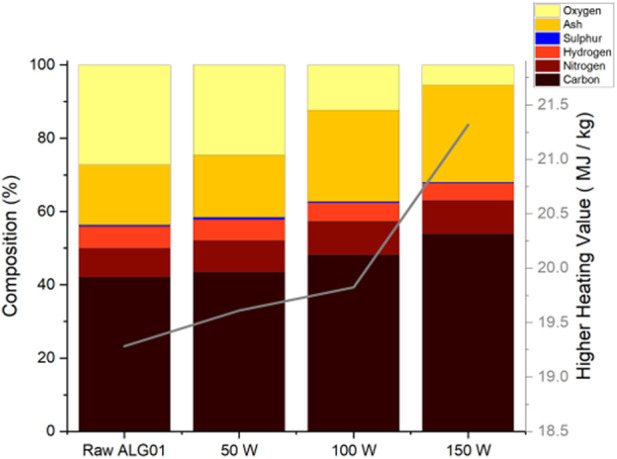
CHNS analysis and ash content of the ALG01 biochars (50–150 W) and raw dried biomass and their calculated HHV (line).

With increased microwave power, the carbon and nitrogen content both increased from 42.14% ± 0.11% to 53.91% ± 3.21% and 7.93% ± 0.24% to 9.21% ± 1.33%, respectively. The hydrogen and oxygen content both decreased from 5.88% ± 0.07% to 4.64% ± 0.25% and 27.20% ± 1.12% to 5.47% ± 2.44%, respectively. Interestingly, the sulphur content increased from 0.39% ± 0.04% (Raw ALG01) to 0.61% ± 0.01% (50 W) before decreasing to 0.25% ± 0.12% (150 W) (Figure 2). This indicates the removal of sulfur within the biogas since the bio-oil fraction also exhibits a decrease in sulfur at higher power (Section 3.4.1.) The higher heating value of the char was calculated using Channiwala and Parikh’s (2002) equation. The HHV of all the biochars was higher that the HHV of the RAW ALG01, which implies that the biochar has undergone energy densification. The highest HHV was achieved by the 150 W biochar (21.32 ± 1.58 MJ/kg). The HHV of the 150 W biochar is equivical to that of sub-bituminous coal (19.31-26.75 MJ/kg), making it a possible replacement for non-renewable solid fuel sources (Carpenter, 1988). The minimal sulfur content is beneficial for fuel applications however the high ash content is likely to cause slag formation within equipment. This can be minimised by washing the biochars to reduce the ash content and increase the HHV values.

The ash content is depicted in Table 2. The ash content was determined by introducing oxygen and a 30 min isothermal hold to the end of the thermogravimetric analysis. The ash predominantly contains non-combustible inorganics. As microwave power increased, the ash content increased from 16.47% ± 1.19% (Raw ALG01) to 26.53% ± 2.07% (150 W). This is to be expected as with the decreased biochar yield, the relative percentage of inorganics will increase.

**TABLE 2:** Ash content of unwashed microwave pyrolysed biochars (50–150 W).

Sample	Ash content (%)
ALG01	16.46
50 W	16.96
100 W	24.92
150 W	26.53

#### 3.2.2 Thermogravimetric analysis (TGA), solid state ^13^C NMR, and FTIR

**FIGURE 3:**
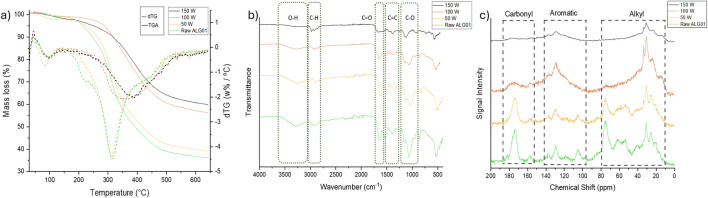
**(A)** TG and DTG thermograms, **(B)** ATR-IR spectra, and **(C)** ^13^C CPMAS NMR of ALG01 biochars (50–150 W) and raw dried biomass.

The TGA and DTG thermograms of the biochars and Raw ALG01 are depicted in Figure 3A. The first peak, which appears as a mass gain, is due to the buoyancy effect. The first mass loss (50 °C–120 °C) is due to the loss of volatiles and residual moisture. The second mass loss (220 °C–320 °C) is due to the decomposition of cellulose and hemicellulose. The 50 W char appears to have undergone rather minimal pyrolysis. There is a minor decrease in the 220 °C mass loss (shoulder) associated with hemicellulose, furthermore, this sample exhibits a minor increase in volatile content in combination this could indicate hemicellulose scissoring. Both the 100 W and the 150 W biochar appear to have undergone increasingly thorough pyrolysis than the 50 W char, the main degradation peak (∼320 °C), associated with cellulose, has shifted from 320 °C to 350 °C and 400 °C for the 100 W and 150 W biochars, respectively. The 100 W char also has a significantly broader DTG peak (300 °C–450 °C) than the 150 W (350 °C–450 °C) which likely indicates some residual cellulose at 100 W. The higher residual mass of these chars also suggests that a significant proportion of thermally unstable functionalities may have already been removed.

Figure 3B displays the ATR-IR spectra of the microwave biochars and the starting material (Raw ALG01). A broad absorbance at 3,300 cm^-1^ and sharp peak at 1,670 cm^-1^ indicate the presence of O-H groups and C=O groups respectively. There is a shift in the relative intensity of the C-O absorbance, with the major peak shifting from 1,072 cm^-1^ in the Raw ALG01 to 1,123 cm^-1^ in the 150 W biochar. With increasing microwave power, the intensity of these peaks decreases, suggesting the deoxygenation and decarboxylation of the saccharide polymers has occurred at the higher microwave powers. This is concurrent with elemental analysis which indicates a loss in oxygen content. The C-H stretch shifts across the three microwave chars, from sp^3^ C-H stretching at 2,898 cm^-1^ (50 W) to higher energy vibrations at 2,924 cm^-1^ (100 W) and further to 2,990 cm^-1^ (150 W). This could indicate degrees of unsaturation forming at 150 W although the stretching frequency is lower than expected for sp^2^ C-H stretching.

From the SS 13C CPMAS NMR (Figure 3C), we can more clearly see the change in functionality experienced by the higher power biochars. Both the Raw ALG01 and the 50 W biochar present very similar spectra although a slight decrease in the peak intensity of the carbonyl carbon environments at ∼175 ppm. Both the 100 W and 150 W biochars experience a very large reduction in the relative intensity of the carbonyl peak, which is further evidence of decarboxylation. The higher power chars (100 W and 150 W) also exhibit an increase in aromatic/alkene carbon environments, and a reduction in oxygenated carbon environments. This further supports FTIR, CHN, and TGA results and suggests that under the sealed system the minimal oxygen content drives the deoxygenation and subsequent decarboxylation of polysaccharide backbones and the reformation of sp^3^ carbon environments into sp^2^ carbon chains or rings.

#### 3.2.3 Scanning electron microscopy (SEM)

**FIGURE 4:**
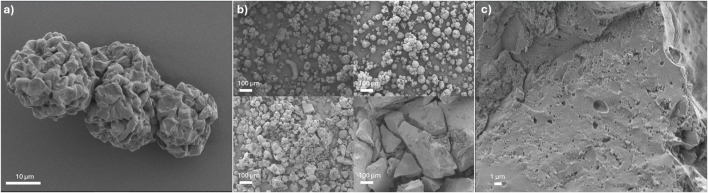
**(A)** ×1,800 magnification image of Raw ALG01. **(B)** Raw dried biomass (top left), 50 W biochar (top right), 100 W biochar (bottom left) and 150 W biochar (bottom right). Images taken at 2 kV, ×160 magnification. **(C)** ×3,700 magnification image of 150 W biochar after washing.

**FIGURE 5:**
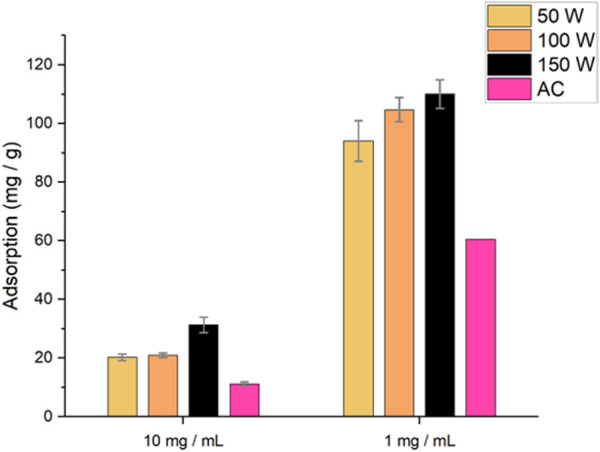
Preliminary copper(II) adsorption capacity of ALG01 biochars and AC at variable doses (1 and 10 mg/mL) from an aqueous copper(II) nitrate solution ([Cu^2+^] 0.655 mg/L).

The SEM images of the biochars and Raw ALG01 can be seen in Figure 4. The Raw ALG01 displays dimpled, ‘golf ball’ like structures which appear to be composed of collapsed cells (10 nm–40 nm) producing the dimpled effect (Figure 4A). The clumping of the cells is likely due to spray drying, whilst the dimpling and smaller cell size is an effect of the drying method and lipid extraction process. The 50 W biochar predominantly consists of the ‘golf ball’ like structures, however, there is indication of ‘golf ball’ melding into singular masses. The 100 W char consists of a mixture of solid charred masses, and conjoined ‘golf balls’ which resemble the Raw ALG01. The 150 W char, however, exhibits none of the biomass characteristics and has formed amorphous brittle structures (Figure 5B). No visible pores were observed on any of the chars before washing to remove the inorganic salts. Only after washing, mesopores became visible on the 150 W char (Figure 5C).

### 3.3 Biochar copper(II) adsorption

A preliminary study investigating the effect of varying biochar concentration of Cu(II) removal from solution was explored (Figure 5). When the biochar was dosed at 10 mg/mL, both the 50 W and 100 W biochars performed similarly, adsorbing ∼20.5 mg/g, whilst activated carbon (AC) absorbed 11.22 ± 0.62 mg/g of copper. The 150 W biochar performed the best, adsorbing 31.26 ± 2.63 mg/g of copper. When dosed at 1 mg/mL, all of the biochars adsorbed a significantly greater amount of copper. This is due to the high saturation of adsorbent sites and a higher concentration gradient of copper (II) from the solution to the biochar. Moreover, at high adsorbent concentrations, the biochar would have a decrease in effective surface area due to the particles aggregating (Mashkoor et al., 2018). The 150 W biochar adsorbed the highest amount of copper(II) (110.00 ± 4.83 mg/g), whilst the 50 W, 100 W and AC adsorbed 94.01 ± 6.88 mg/g, 104.65 ± 4.13 mg/g and 60.50 ± mg/g respectively. Tukey’s HSD post hoc test showed that at 1 mg/mL, the 150 W biochar showed a significant increase in adsorption capacity whilst at 10 mg/mL, there was a significant difference between the 50 W and 150 W adsorption capacities. AC showed a significantly lower adsorption capacity than all biochars (Supplementary Table S5). It is not surprising that the 150 W biochar adsorbed the highest amount of copper(II), as the formation of mesopores on the surface of the char will greatly increase the surface area, allowing a larger amount of copper(II) to be adsorbed.

### 3.4 Bio-oil analysis

#### 3.4.1 CHNS and HHV

**FIGURE 6:**
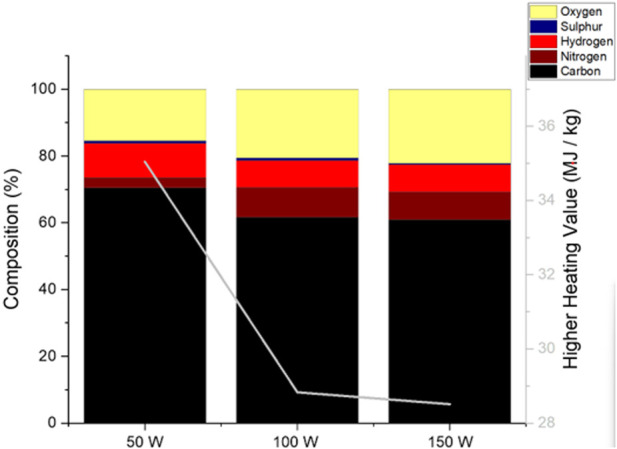
CHNS analysis of the ALG01 bio-oils (50–150 W) and their calculated HHV (line).

The CHNS content and HHV of the bio-oils is depicted in Figure 6. Surprisingly, the C and H content of the 50 W bio-oil was the highest at 70.46% ± 5.01% and 10.21% ± 0.11% respectively, whilst also having the lowest nitrogen (3.09% ± 0.03%) and oxygen (15.55% ± 5.05%) content. The carbon, nitrogen, hydrogen and oxygen content of the 100 W and 150 W bio-oils are relatively similar, with exception of the sulfur content, which is 2x greater in the 100 W bio-oil than the 150 W bio-oil. The 50 W bio-oil has the highest HHV (35.04 ± 2.40 MJ/kg), which is due to its very high carbon and hydrogen content. The HHV of both the 100 W (28.83 ± 1.34 MJ/kg) and 150 W (28.51 ± 0.09 MJ/kg) bio-oil is again very similar. The 50 W bio-oil would be the best option for a fossil fuel replacement as it has the highest HHV. Moreover, due to the low nitrogen and sulfur content, the amount of harmful NO_x_ and SO_x_ produced would be minimal.

#### 3.4.2 ATR-IR

**FIGURE 7:**
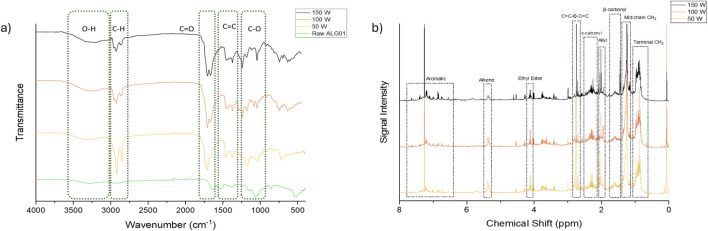
**(A)** ATR-IR spectra of the ALG01 bio-oils and the raw dried biomass. **(B)** 1H NMR of ALG01 bio-oils dissolved in chloroform-D.

The ATR-IR of the bio-oils and Raw ALG01, for comparison, are depicted in Figure 7A. All of the bio-oils exhibit a broad O-H absorbance at 3,260 cm^-1^ and a strong absorbance at both 1720 cm^-1^ (C=O) and 2,920 cm^-1^ (C-H). The O-H stretch is synonymous with that of carboxylic acid bonding. The C-H absorbances between 2,800 cm^-1^ and 3028cm ^-1^ are representative of CH_2_ and CH_3_ bonding in long chain fatty acids. The peaks at 2,853 cm^-1^ and 2,924 cm^-1^ represent CH_2_, whilst the peaks at 2,870 cm^-1^ and 2,961 cm^-1^ represent CH_3_. The ratio of CH_2_:CH_3_ peaks in the 50 W bio-oil is much greater than that of the 100 W and 150 W bio-oils. Furthermore, the ratio of the peaks at 1708 cm^-1^ and 1728 cm^-1^ varies with increasing microwave power, with the former being more prevalent in the 50 W bio-oil and the latter being more prevalent in the 100 W bio-oil. Interestingly, these peaks in the 150 W bio-oil exhibit a similar intensity.

Proton NMR analysis (1H) of the bio-oil samples are shown in Figure 7B. The fatty acid (FA)peaks were identified by comparison to literature (de Jesus et al., 2015). The peaks around 7 ppm indicate the presence of aromatic protons. The peak at 5.35 ppm represents the protons present in C=C. The quartet at 4.1ppm peak represents the CH_2_ of the ethyl ester form of the fatty acid, which confirms the presence of fatty acid ethyl esters (FAEEs) found by GC-MS. The latter are present due to the EPA extraction process which involves transesterication with ethanol. The peak at 2.76 ppm represents the proton of a CH_2_ between 2 adjacent C=C, which would be present in both EPA and linoleic acid. The peaks around 2.29 ppm represent the α-carbonyl protons from the FAs and FAEEs. The peaks around 2.04 ppm represent the allyl protons, which could be attributed to EPA, linoleic acid, neophytodiene and phytol. The peaks around 1.6 ppm represent the β-carbonyl protons. The peak at 1.25 ppm represents the mid-chain CH_2_ protons. The peaks between 1.0 and 0.85 ppm represent the protons on the terminal carbon of the FA chains. This is concurrent with ^13^C NMR spectra (Supplementary Figure S1). All of the bio-oils exhibit expected peaks in the carbonyl and alkyl region, due to the high lipid content of the oil. These peaks also match those found in literature. Moreover, the peaks in the region of 100–140ppm, indicate the presence of aromatic compounds.

#### 3.4.3 GC-MS/FID

**FIGURE 8:**
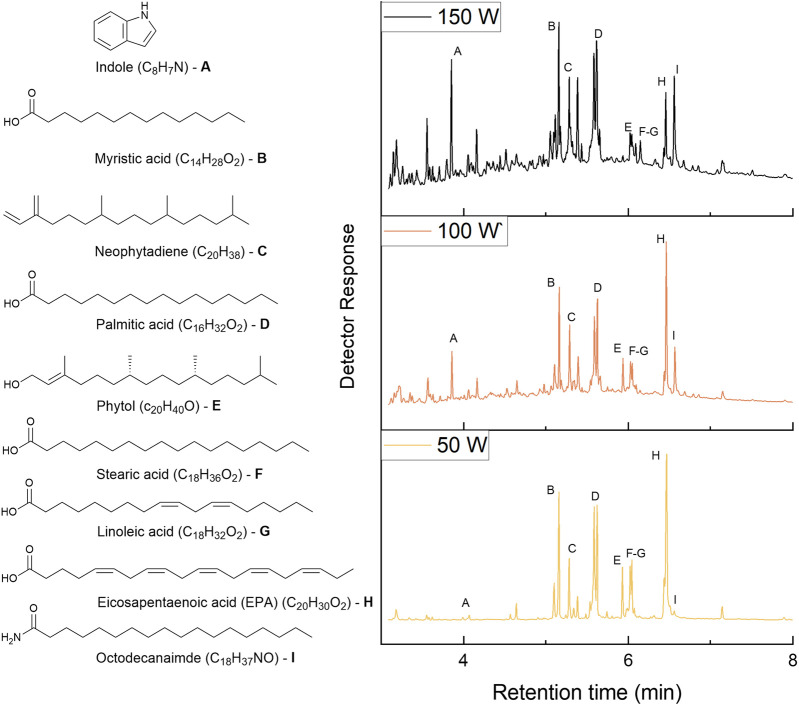
GC-MS chromatograms of ALG01 bio-oils (right) with the corresponding structures (left).

GC-MS was used to identify the major compounds present in the bio-oils (Figure 8). All of the bio-oils consisted predominantly of long chain fatty acids. Indole was found in all 3 bio-oils with an elevated yield found with increasing microwave power. This is confirmed with the increase in aromatics found in the ^13^C NMR. Indole is most likely a product from homolysis of tryptophan (Huang et al., 2017). The ratio of octodecanamide to stearic acid increased drastically with increased microwave power. This is most likely due to the reaction of an amino group with stearic acid (Huang et al., 2017). Similarly, the ratio of phytol, derived from chlorophyll-a degradation, to neophytadiene decreased with increased microwave power as a result of dehydration. Moreover, due to cracking, the ratio of small molecular weight molecules to long chain fatty acids increased as well.

In comparison to the literature, the achieved yields of both biochar and biogas are similar whilst the bio-oil yield is much lower (Table 3). This is due to the condensation and polymerisation of the bio-oil onto the biochar’s surface, as pyrolysis was conducted in a closed system, leading to increased biochar:bio-oil yield (Sun et al., 2024). Currently, microwave pyrolysis conducted in the literature only uses an open system with either a constant flow of inert gas or under vacuum, both of which remove volatiles, preventing the bio-oil from condensing and contributing to the formation of the biochar. When accounting for the microwave’s energy efficiency (55%–70% - Priecel and Lopez-Sanchez, 2019), the use of a vacuum pump (<0.25 kW - S-Lab audits, 2011) and/or compressed gas (0.31–0.63 kWh/kg N_2 (g)_ - Syakdani. 2019), our microwave set up uses substantially less energy than those reported in the literature. It is also important to note that the current literature focuses primarily on the production of bio-oils and/or biogas, while an alternative application for the biochar has, until now, not been explored.

**TABLE 3:** Comparison of reaction conditions, yields and HHVs from our study to literature.

Biomass	Conditions	Biochar yield (%)/HHV (MJ/kg)	Bio-oil yield (%)/HHV (MJ/kg)	Biogas yield (%)	Assumed energy consumption (W)^c^	Ref
Polyculture	300–900 W, 5–30 min, N_2_ (purge – 1 L/min, 2 min. Run - 100 mL/min)	11.6–69.2/28.3^b^	—	—	40.7–721.4	Mohit et al (2023)
*Scenedesmus almeriensis*	250–950 W, <140 min, He (20 mL/min), 3:7 biochar:biomass	26.9–32.4	15.4–23.1	44.5–57.5	-	Beneroso et al (2013)
*Chlorella sp.*	500–1250 W, 20 min, N_2_ (500 mL/min), 1:5 biochar:biomass	25.6–28.4	17.8–28.6/30.2^a^	24.2–34.8	269.4–669.4	Du et al (2011)
*Chlorella vulgaris*	750–2250 W, 32 min, N_2_ (purge −300 mL/min, 20 min. Run – 300 mL/min), 5%–30% catalyst (activated carbon, SiC, CaO)	∼25–90	1.0–35.8	10–52.4	640.0–1920.0	Hu et al (2012)
*Chlorella sp.*	600 W, 20 min, vacuum, 1:5 CaO: biomass	∼51–59	18.6–24.4/43^b^	∼22–24	320.1	Qadariyah et al (2021)
*Chlorella sp.*	1250 W, 20 min, vacuum, 12.5–25 wt% activated carbon:biomass	20.6	56.7/43^b^	22.7	666.7	Mahfud et al (2024)
*C. vulgaris*	390–700 W,10 min, N_2_ (purge - 400 mL/min, 30 min. Run – 400 mL/min), 1:5–3:5 lignite char/Fe_2_O_3_:biomass	∼23–25	∼48–56	∼21–30	104.0–186.7	Wang et al (2015)
Defatted ALG01	50–150 W, 20 min, closed vessel	55.67–86.57/21.3^a^	1.0–5.19/35.0^a^	39.61–12.5	26.7–80.0	This study

^a^Calculated using given CHNS and Channiwala’s equation.

^b^Calculated using bomb calorimetry.

^c^Assuming 62.5% microwave efficiency, 0.47 kWh/kg of gaseous N_2_, vacuum pump running at 0.1875 kW.

## 4 Conclusion

Our study demonstrates that the use of a sealed vessel microwave can pyrolyze microalgae at much lower powers, offering a more efficient alternative to traditional microwave pyrolysis. The produced biochars were shown to increase in HHV from 19.28 ± 0.13 MJ/kg (Raw ALG01) to 21.32 ± 1.58 MJ/kg (150 W) showing that the biochars had undergone energy densification from the raw dried biomass. Moreover, the biochars all showed a decrease in functionality with increased microwave power, which indicates a higher level of pyrolysis. Preliminary copper(II) adsorption was performed with the biochars and AC. The biochars and AC showed an increased copper(II) adsorption capacity when dosed at 1 mg/ml as opposed to 10 mg/mL, with the maximum capacity adsorbed being 110.00 ± 4.83 mg/g by the 150 W biochar. The produced bio-oils were shown to be rich in long chain fatty acids. The HHV of the bio-oils decreased with increased microwave power, which is due to the cracking of the long chain fatty acids. Thus, the use of low power as opposed to high power and/or high temperature conventional pyrolysis yields interesting products that can be used as potential platform chemicals, adsorbents for critical element recovery and as a potential solid biofuel. We acknowledge certain limitations of this study. Thus, in future work, the effect of residency time should be investigated. Co-feedstock pyrolysis should be investigated for any synergistic effects. Moreover, the copper(II) adsorption capacity of the biochars should be fully explored as well as a competitive metal adsorption study. The work should be conducted at Technology Readiness Levels (3–5) so that meaningful LCA and TEAs can be conducted to determine the industrial applicability of the process. The application of the biochar as a potential solid fuel is limited because the HHV is similar to that of low grade coal and has a relatively high ash content. The latter may be improved with washing to remove minerals and salts.

## Data Availability

The original contributions presented in the study are included in the article/Supplementary Material, further inquiries can be directed to the corresponding author.
