# Supplementary material for: Evaluating the products of low power, sealed vessel microwave pyrolysis of microalgae
Source: Front Chem. 2026 Mar 12;14:1735803. doi: 10.3389/fchem.2026.1735803 (PMC13018125; doi:10.3389/fchem.2026.1735803)
Supplement: Supplementary file 1 [file DataSheet1.docx]

Supplementary Material

# Supplementary Data

Supplementary Material should be uploaded separately on submission. Please include any supplementary data, figures and/or tables.

Supplementary material is not typeset so please ensure that all information is clearly presented, the appropriate caption is included in the file and not in the manuscript, and that the style conforms to the rest of the article.

# Supplementary Figures and Tables

For more information on Supplementary Material and for details on the different file types accepted, please see [here](https://www.frontiersin.org/guidelines/author-guidelines#supplementary-material).

## Supplementary Figures

**Supplementary Figure 1:** ^13^C NMR spectra of the ALG01 bio-oils (2048 scans).

Supplementary Table 1: Tukey HSD post hoc statistical analysis of maximum temperature and maximum pressure. Significances <0.05 are highlighted in bold italics

| Sample | Sample comparison | Sig. | |
| --- | --- | --- | --- |
|  |  | Maximum Temperature | Maximum Pressure |
| 50 W | 100 W | *0.001* | 0.094 |
|  | 150 W | *0.000* | *0.006* |
| 100 W | 50 W | *0.001* | 0.094 |
|  | 150 W | *0.000* | 0.094 |
| 150 W | 50 W | *0.000* | *0.006* |
|  | 100 W | *0.000* | 0.094 |

Supplementary Table 2: Tukey HSD post hoc statistical analysis of biochar, bio-oil and biogas yields. Significances <0.05 are highlighted in bold italics

| Sample | Sample comparison | Sig. | | |
| --- | --- | --- | --- | --- |
|  |  | Biochar Yield | Bio-oil Yield | Biogas Yield |
| 50 W | 100 W | *0.000* | *0.020* | *0.000* |
|  | 150 W | *0.000* | *0.034* | *0.000* |
| 100 W | 50 W | *0.000* | *0.020* | *0.000* |
|  | 150 W | 0.271 | 0.897 | *0.006* |
| 150 W | 50 W | *0.000* | *0.034* | *0.000* |
|  | 100 W | 0.271 | 0.897 | *0.006* |

Supplementary Table 3: Tukey HSD post hoc statistical analysis of CHNSO, ash content and HHVs of biochars. Significances <0.05 are highlighted in bold italics

| Sample | Sample comparison | Sig. | | | | | | |
| --- | --- | --- | --- | --- | --- | --- | --- | --- |
|  |  | Carbon | Hydrogen | Nitrogen | Sulphur | Oxygen | Ash | HHV |
| 50 W | 100 W | 0.09 | 0.08 | 0.66 | 0.14 | *0.00* | *0.00* | 0.12 |
|  | 150 W | *0.00* | *0.02* | 0.68 | 0.08 | *0.00* | *0.00* | *0.01* |
| 100 W | 50 W | 0.09 | 0.08 | 0.66 | 0.14 | *0.00* | *0.00* | 0.12 |
|  | 150 W | *0.04* | 0.35 | 1.00 | 0.83 | *0.02* | 0.61 | 0.07 |
| 150 W | 50 W | *0.00* | *0.02* | 0.68 | 0.08 | *0.00* | *0.00* | *0.01* |
|  | 100 W | *0.04* | 0.35 | 1.00 | 0.83 | *0.02* | 0.61 | 0.07 |

Supplementary Table 4: Tukey HSD post hoc statistical analysis of CHNSO content and HHVs of bio-oils. Significances <0.05 are highlighted in bold italics

| Sample | Sample comparison | Sig. | | | | | |
| --- | --- | --- | --- | --- | --- | --- | --- |
|  |  | Carbon | Hydrogen | Nitrogen | Sulphur | Oxygen | HHV |
| 50 W | 100 W | *0.028* | *0.009* | *0.000* | 0.938 | 0.194 | *0.006* |
|  | 150 W | *0.023* | *0.010* | *0.001* | *0.003* | 0.110 | *0.007* |
| 100 W | 50 W | *0.028* | *0.009* | *0.000* | 0.938 | 0.194 | *0.006* |
|  | 150 W | 0.915 | 0.943 | 0.224 | *0.003* | 0.780 | 0.970 |
| 150 W | 50 W | *0.023* | *0.010* | *0.001* | *0.003* | 0.110 | *0.007* |
|  | 100 W | 0.915 | 0.943 | 0.224 | *0.003* | 0.780 | 0.970 |

Supplementary Table 5: Tukey HSD post hoc statistical analysis of copper(II) adsorption capacity. Significances <0.05 are highlighted in bold italics

| Sample | Sample comparison | Sig. | |
| --- | --- | --- | --- |
|  |  | 10 mg / ml | 1 mg / ml |
| 50W | 100W | 0.939 | 0.085 |
|  | 150W | *0.000* | *0.013* |
|  | AC | *0.000* | *0.001* |
| 100W | 50W | 0.939 | 0.085 |
|  | 150W | *0.000* | 0.543 |
|  | AC | *0.000* | *0.001* |
| 150W | 50W | *0.000* | *0.013* |
|  | 100W | *0.000* | 0.543 |
|  | AC | *0.000* | *0.001* |
| AC | 50W | *0.000* | *0.001* |
|  | 100W | *0.000* | *0.001* |
|  | 150W | *0.000* | *0.001* |

*Supplementary Figure 2: Copper(II) calibration curve calculated using copper(II) nitrate solutions (0, 100, 500, 1000, 1500, 2500, 3500 and 5000 mg / L)*
